# AI-assisted image analysis and physiological validation for progressive drought detection in a diverse panel of *Gossypium hirsutum* L.

**DOI:** 10.3389/fpls.2023.1305292

**Published:** 2024-02-21

**Authors:** Vito Renó, Angelo Cardellicchio, Benjamin Conrad Romanjenko, Carmela Rosaria Guadagno

**Affiliations:** ^1^ Institute of Intelligent Industrial Technologies and Systems for Advanced Manufacturing, National Research Council of Italy (CNR STIIMA), Bari, Italy; ^2^ Department of Botany, University of Wyoming, Laramie, WY, United States; ^3^ Science Initiative, University of Wyoming, Laramie, WY, United States

**Keywords:** thermal imaging, drought, plant phenotyping, machine learning, leaf classification, artificial intelligence

## Abstract

**Introduction:**

Drought detection, spanning from early stress to severe conditions, plays a crucial role in maintaining productivity, facilitating recovery, and preventing plant mortality. While handheld thermal cameras have been widely employed to track changes in leaf water content and stomatal conductance, research on thermal image classification remains limited due mainly to low resolution and blurry images produced by handheld cameras.

**Methods:**

In this study, we introduce a computer vision pipeline to enhance the significance of leaf-level thermal images across 27 distinct cotton genotypes cultivated in a greenhouse under progressive drought conditions. Our approach involved employing a customized software pipeline to process raw thermal images, generating leaf masks, and extracting a range of statistically relevant thermal features (e.g., min and max temperature, median value, quartiles, etc.). These features were then utilized to develop machine learning algorithms capable of assessing leaf hydration status and distinguishing between well-watered (WW) and dry-down (DD) conditions.

**Results:**

Two different classifiers were trained to predict the plant treatment—random forest and multilayer perceptron neural networks—finding 75% and 78% accuracy in the treatment prediction, respectively. Furthermore, we evaluated the predicted versus true labels based on classic physiological indicators of drought in plants, including volumetric soil water content, leaf water potential, and chlorophyll *a* fluorescence, to provide more insights and possible explanations about the classification outputs.

**Discussion:**

Interestingly, mislabeled leaves mostly exhibited notable responses in fluorescence, water uptake from the soil, and/or leaf hydration status. Our findings emphasize the potential of AI-assisted thermal image analysis in enhancing the informative value of common heterogeneous datasets for drought detection. This application suggests widening the experimental settings to be used with deep learning models, designing future investigations into the genotypic variation in plant drought response and potential optimization of water management in agricultural settings.

## Introduction

Climate change is exerting a profound impact on global crop production, primarily driven by the escalating variability in precipitation patterns and the increased occurrence of droughts (IPCC, 2022). These shifts in water availability have far-reaching consequences, affecting the productivity, quantity, and quality of all agricultural crops, including those essential for anthropic use.

One such crop is *Gossypium hirsutum* L., important for its significant contributions to fiber production, seed oil extraction, and livestock fodder. Thriving in arid environments where water resources are already limited, this species necessitates a substantial volume of annual water (60–120 cm) to support its robust growth (Wegier et al., 2016; Khan et al., 2020). With 25 million tons of fiber produced per year and an economic impact exceeding 600 billion dollars, cotton plays a pivotal role in supplying over 80% of the global natural fiber demand, underscoring its critical importance to both individuals and global economies (Townsend, 2020). In recent years, the production of this crop has been decreasing due to more severe weather events (Meyer et al., 2023), and projections suggest that the world cotton production may struggle to meet the burgeoning demand in the next decades (Li et al., 2021). However, a silver lining is represented by the substantial genetic diversity inherent within this species constituting an unprecedented avenue for the selection, breeding, and cultivation of varieties that are inherently better equipped to endure and thrive amidst increasing climatic pressures.

Plant phenotyping consistently applies image processing (IP) techniques (either classical or modern ones to data acquired from visible, infrared, and hyperspectral cameras, showing the potential to enable for non-destructive, high-throughput detection and selection of desirable traits across different temporal and spatial scales (Zhao et al., 2019). Thermal imaging, also known as infrared thermography, is a powerful and non-invasive technique that has found widespread relevance in recent years to assess canopy temperature and their responses to both abiotic and biotic stressors, from salt stress, heat, and drought stress to bacterial and fungal infections (Pineda et al., 2021). The analysis of canopy temperatures has been connected to traditional physiological measurements—leaf water potential, gas exchange, and chlorophyll *a* fluorescence (Cohen et al., 2005; Casari et al., 2019)—and utilized to screen for genotypic variation across several species (Casari et al., 2019; Bhandari et al., 2021; Ferguson et al., 2021). The processing of thermal images usually starts by separating the canopy impression from the background pixels that may include soil particles and other structures. This initial pixel exclusion process can be completed through a variety of different approaches: manual isolation of the canopy and leaf via polygon selection, gray scaling, image segmentation, two-means clustering, and bimodal peak detection (Mohanty et al., 2016; Prakash et al., 2021; Stutsel et al., 2021; Sakurai et al., 2023). Despite the utilized methodology, the postprocessing times for the analysis of thermal images are usually long and often affected by the low resolution of the images (Kohin and Butler, 2004).

To cope with the constraints imposed by traditional IP methods, over the last few years, the scientific community has largely adopted machine learning (ML) and, particularly, deep learning (DL) techniques to deal with data acquired by plant phenotyping platforms or, more in general, from high-throughput measurements (Solimani et al., 2023). These algorithms can also represent a great opportunity to implement the postprocessing of thermal images captured with handheld cameras and indeed increase their final throughput. ML algorithms have already been used to analyze thermal images, specifically to enhance stomatal count, surface recognition, and crop disease classification (Cho et al., 2018; Ferguson et al., 2021; Pignon et al., 2021; Batchuluun et al., 2022). Three different ML algorithms, namely, random forest, multivariate linear regression, and gradient boosting, were previously used to correlate thermal data—acquired by thermal IR images—to environmental drivers, such as solar radiation, air temperature, relative humidity, and wind speed, to assess the relationship between the stomatal conductance in crop canopies and changes in environmental factors (Zhao et al., 2021). Another approach consisted of two models based on variations for decision trees used to define a relationship between the regression of thermal indexes for droughted and well-watered scenarios of vineyard crops (Gutiérrez et al., 2018). DL approaches have also been proposed by developing a custom architecture based on convolutional neural networks (CNNs) to classify five different crop diseases and defects (e.g., blast, bacteria leaf blight, leaf folder) (Batchuluun et al., 2022). In that case, the model was first trained on the Paddy crop dataset and then refined on a new empirical dataset consisting of 4,720 images. The results were further investigated by using class activation maps to highlight the parts of the image that were considered relevant by the network to achieve the classification result. Finally, indexes of classification, such as the crop water stress index (CWSI), have been computed to distinguish between droughted and well-watered crops. Despite these significant IP applications to thermal images and the correlations to different physiological indicators from various crops, models that use thermal crop response to water stress across extreme genotypes using deep learning are scarce (Berni et al., 2009; Pratap et al., 2019).

Here, we provide an evaluation of the response of different genotypes to different levels of water limitations, from mild to severe drought. First, we screened a panel of 27 geographically different genotypes (**Supplementary Figure S2**) in the species *Gossypium* for their response to water limitation using a handheld IR camera. These imaging data were used as the basis to develop a hybrid IP/ML processing software pipeline, which used IP techniques to extract the region of interest from each leaf and then feed a statistically enhanced ML algorithm to predict the leaf water status, as either well-watered (WW) or subjected to dry-down (DD) at two different times during the complete water withholding (mild and severe drought). Finally, we coupled additional leaf-level physiological measurements, such as water potential and chlorophyll *a* fluorescence to the IP/ML analysis, providing a meaningful interpretation of the modeled results.

## Materials and methods

### Plant materials

**Figure 1:**
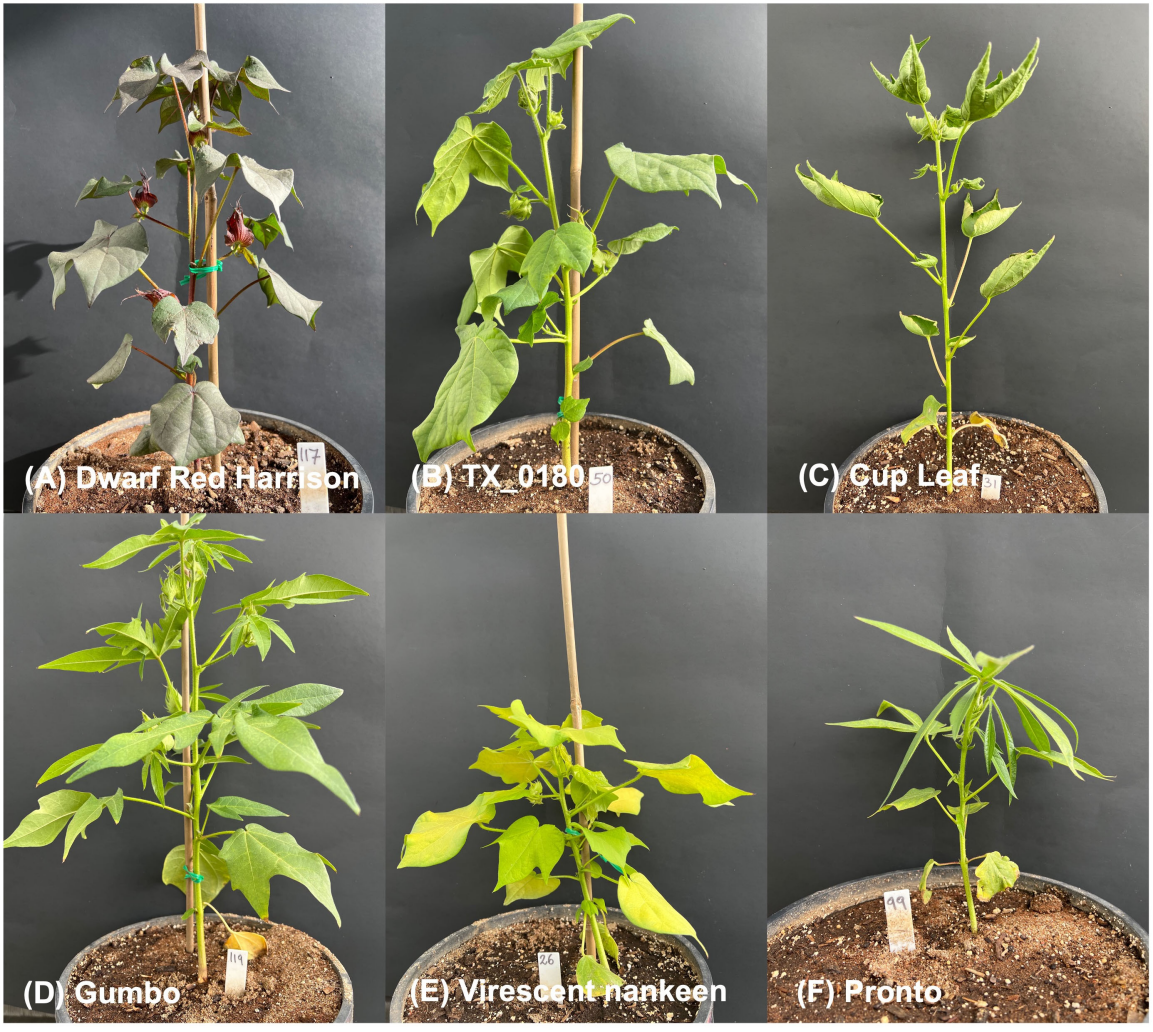
*In-vivo* pictures of extreme genotypes in the cotton panel. Striking examples of plant architecture, leaf types, and coloration differences in the experimental panel. Red/dark green medium size leaf for *Red Dwarf Harrison* **(A)**; large, with low venation leaves in *TX_180* **(B)**; inverted margins for the leaves of *Cup Leaf* **(C)**; trilobate morphology for *Gumbo* leaves **(D)**; pale green and short overall plant size for *Virescent nankeen* **(E)**; and short-overall plant size and okra-like leaves for *Pronto* **(F)**. All plants were WW and imaged on the same day (70 DAS).

A panel of 27 different genotypes was utilized for the experiment, and all seeds were obtained from the USDA Germplasm Collection. Genotypes originate from Australia, China, Guatemala, Mexico, Trinidad and Tobago, and the USA, covering all four zones that have the highest production of cotton in the world (Wendel et al., 2009). Aside from being geographically diverse, the selected genotypes also span a large range in leaf size, plant and leaf architecture, and coloration (**Figure 1**). This extreme genotypic variation inevitably affects the physiology of these genotypes, including their water status and their ability to maintain leaf turgor despite water limitations (e.g., large versus small leaves, significantly impacting transpiration rates) making the panel of choice perfectly suited for testing our pipeline, due to expected great variation in the thermal features of the leaves under progressive drought.

### Growth conditions

The cotton panel was grown in a greenhouse research bay of the Plant Growth & Phenotyping Facility at the University of Wyoming (Laramie, Wyoming, USA) for a total of 122 days from seed to seed, planting to harvesting, during the winter of 2022–2023. The cotton panel, 27 genotypes × 3 replicates each, was grown following a random block design, and the greenhouse environmental conditions were controlled by state-of-the-art climate control systems (Argus, British Columbia, Canada). Temperature was set to 27°C ± 3°C/26°C ± 3°C (day/night), and relative humidity was between 10% and 30%. Additional lighting was given by a four-channel Heliospectra growth light system (Heliospectra AB, Gothenburg, Sweden). The intensity of the Elixia LED channels was set as follows: 450 nm (blue) at 500 units, 660 nm (red) at 500 units, 735 nm (far-red) at 500 units, and the white 5,700K LED channel at 1,000 units. All intensities are reported as 0–1,000 units corresponding to 0%–100% of max LED output as for the Heliospectra manual. The photoperiod was 14/10 (D/N), 0600h–0800h; the highest recorded photosynthetically active radiation (PAR) was 1,600 μmol photons m^−2^ s^−1^ with the sensor located in the middle of the canopy. Aside from the OMNI sensors from Argus, the environmental conditions were also tracked using CR1000 Data Logger (Campbell Scientific Inc. Logan, UT, United States) monitoring: air temperature and relative humidity HMP45AC (VAISALA, Vantaa, Finland); PAR, LI-COR Quantum (LI-COR, Lincoln, NE, United States); and soil moisture, Delmhorst GB-1 (Delmhorst Instrument Co., Towaco, NJ, United States). Sensors were spaced across the entire area covered by canopy in the ~40-m/420-ft^2^ greenhouse bay.

### Experimental design

**Figure 2:**
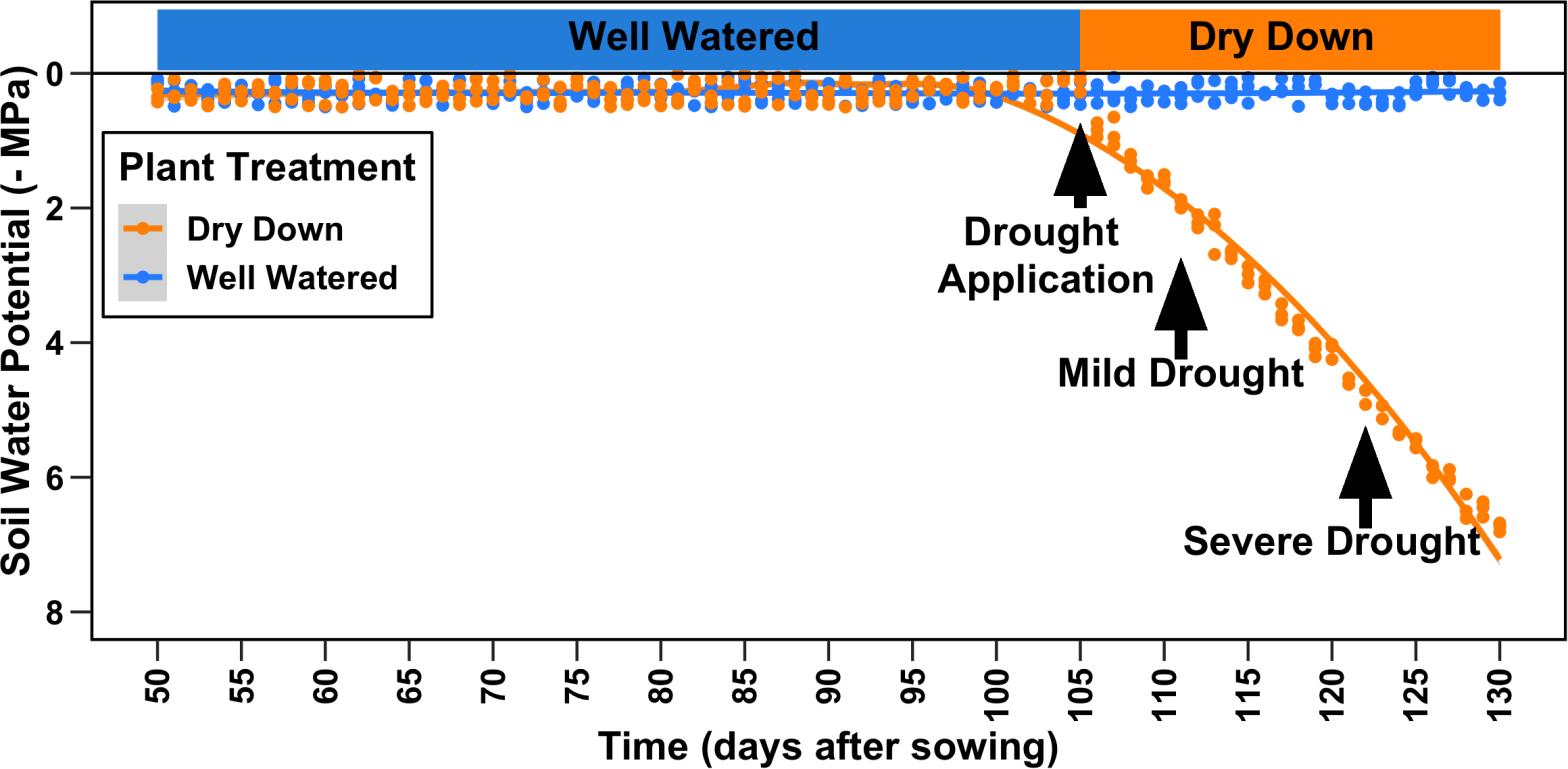
Experimental design. A panel of 27 diverse genotypes of cotton was grown for a total of 122 days after sowing (DAS). All plants were watered at saturation until 105 DAS when drought was applied as complete water withholding for a subset of plants (dry-drown). All data presented in the manuscript were collected at 110 DAS (mild drought) and 121 DAS (severe drought).

One seed per pot (10 quarts/11 L in volume) was sown in a substrate made up of sand (80% v/v; Premium Play Sand, Quickrete, Atlanta, GA), fritted clay (10% v/v; Greens Grade, Buffalo Grove, IL), and organic soil mix (10% v/v; Miracle-Gro moisture control Potting Mix, Marysville, OH) amended with ½ tablespoon of Osmocote 16–6–12 fertilizer (Scotts, Marysville, OH). Sown seeds were covered and placed centrally in the pot at a depth of ~½ inch/1.2 cm and covered in vermiculite to aid in the germination. Plants were hand-watered with reverse osmosis (RO) water daily to maintain soil field capacity and a soil water potential close to saturation until 105 days after sowing (DAS) when all genotypes and replicates had at least 50% of opened flowers (**Figure 2**). At 106 DAS, two randomly chosen replicates for each genotype were subjected to complete water withholding for the rest of the experiment forming the dry-down cohort of plants (DD), while one replicate per genotype was maintained at the daily watering regime in the well-watered (WW) cohort. All physiological measurements occurred at two points in time, at 110 DAS (mild drought) and 121 DAS (severe drought), after 4 and 14 days of uninterrupted progressive drought, respectively.

### Leaf-level physiological measurements

On measurement days, chlorophyll *a* fluorescence was measured on two separate fully developed leaves of the mid-canopy with a handheld fluorometer (FluorPen FP100, Photon System Instruments, Drásov, Czech Republic). Measurements of photosystem II efficiency were taken using a saturation pulse that was applied (1,500 μmol photons m^−2^ s^−1^) to measure *Fv/Fm* or *Fv′/Fm′* on dark- or light-acclimated leaves, respectively (Murchie and Lawson, 2013). During the same measurement days, one fully developed leaf per plant was also harvested and used to measure leaf water potential (PMS Instrument Company, Albany, OR, United States). Soil moisture measurements were also taken using a HydroSense II (Campbell Scientific Inc., Logan, UT, United States). Leaf water potential, chlorophyl *a* fluorescence, and soil moisture measurements were taken over a 24-h time course during the hours of 10:00–12:00 h, 16:00–18:00 h, and 22:00–24:00 h. After the start of the dry-down, all physiological measurements were taken during the hours of 04:00–06:00 h (predawn) and 11:00–13:00 h (midday).

### Thermal imagery collection

**Figure 3:**
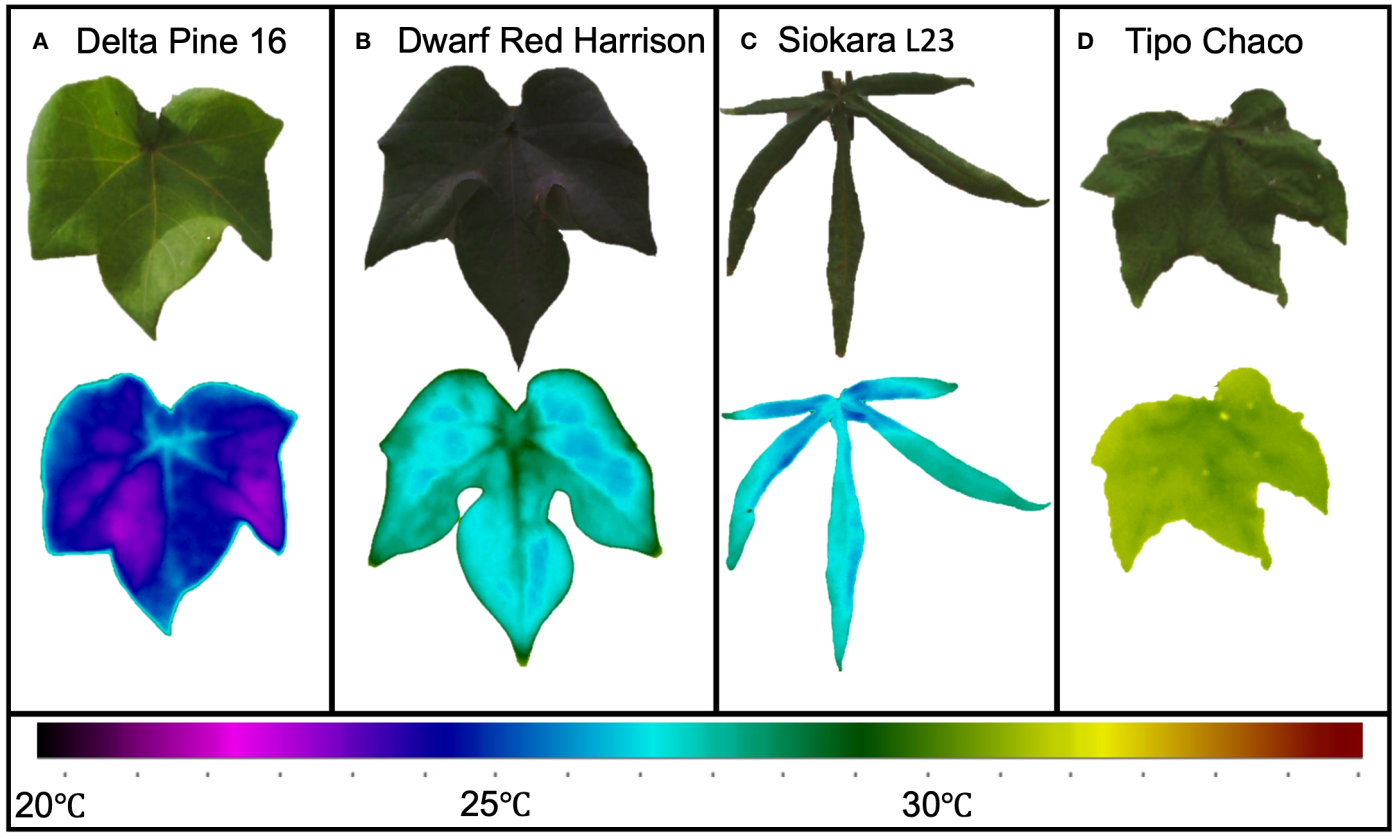
Leaf thermal variation in extreme cotton genotypes. Bright green, large leaf from *Delta Pine 16* **(A)**; red/dark green medium size leaf from *Red Dwarf Harrison* **(B)**; okra-like leaf from *Siokara L23* **(C)**, and dark green medium to large leaf from *Tipo Chaco* **(D)**.

Thermal images were taken using a handheld FLIR Thermal Camera T560, 640 × 480 pixel resolution, wide angle lens *f* = 10 mm (FLIR Systems Inc., Wilsonville, OR, United States). Fully developed leaves near the top of the canopy were chosen for imaging, and one leaf per replicate plant across all genotypes and treatment was imaged at the same time as the other leaf-level physiological measurements. A white paper backdrop was placed directly behind the leaf, and an image was taken holding the camera objective facing both the leaf and backdrop to allow for a full frontal view of the images (**Figure 3**). Image parameters were set using leaf emissivity, 0.95, and with focus regulation (Buitrago et al., 2016). A total of 648 images was made up from two images per leaf, from three replicate plants for 27 genotypes at two times of the day (predawn and midday) and at two drought treatments. After initial QC, the final dataset used for the ML analysis was a balanced dataset of 419 images between WW and DD. All thermal images were converted to *CSV* format using FLIR Thermal Studio.

### Data analysis

Physiological data were processed using Excel and R 4.3.1 (R Core Team, 2013) with packages dplyr (Wickham et al., 2023) and tidyverse (Wickham et al., 2019). The presented graphs were generated using the packages ggplot2 (Wickham, 2016) and ggrepel (Slowikowski, 2023).

### Hybrid IP/ML software pipeline for thermal data

The hybrid IP/ML pipeline used in this work is summarized in **Figure S2**, and it includes the following computational steps.

**Figure 4:**
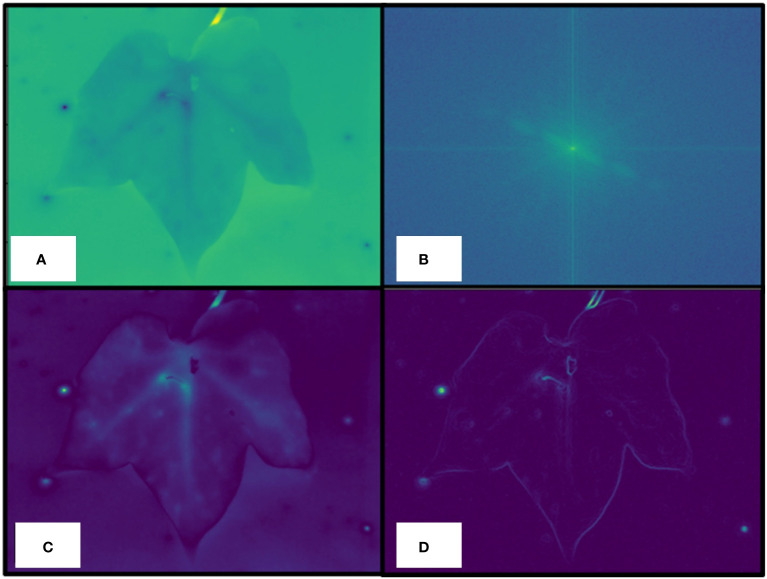
Data preprocessing details. Raw thermal image **(A)**, a fast Fourier transform (FFT) of the raw thermal image is computed to keep all the FFT pixels whose value is less than the continuous component, considered as the central, brighter, pixel **(B)**, inverse transform application **(C)**, computed gradient of the filtered image **(D)** used to compute the actual leaf mask.

1. *Data parsing*: First, raw format data exported by the FLIR thermal camera were parsed by a specific software routine to store the data in an interoperable format such as comma-separated value (CSV) files. Each one of these files held two separate representations, that is, a thermal representation, where each pixel represented a thermal value stored as a floating point number, and an RGB value, used for visualization purposes.

2. *Data preprocessing*: After the parsing step, a preprocessing step aimed at obtaining a high-pass-filtered version of the raw thermal image, as well as its gradient, was performed. To this end, the fast Fourier transform (FFT) of the raw thermal image was first computed (**Figure 4A**). Then, a binary mask was computed to keep all the FFT pixels whose value was less than the continuous component, considered as the central, brighter, pixel (**Figure 4B**). Then, the inverse transform was applied, as shown in **Figure 4C**. Finally, the gradient of the filtered image was computed (**Figure 4D**) and used to compute the leaf mask in the next step.

3. *Leaf mask computation*: The computation of the leaf mask is performed starting from the gradient obtained during the preprocessing step. First, the first quartile $q_{1}$ and the third quartile $q_{3}$ of the values of the gradient image are computed. Then, the interquartile range $IQR=q_{3}-q_{1}$ is the used to compute a threshold $thr_{dw}=q_{1}-1.5\cdot IQR$ . Let δ be the gradient image; a binary mask M is then obtained according to the following binarization logic:


$$M\left(i,j\right)=\{\begin{array}{c} 1,\;if\;\delta \left(i,\;j\right)\;\vee \delta \left(i,j\right)<thr_{dw}\;\\ 0,\;otherwise \end{array}$$

**Figure 5:**
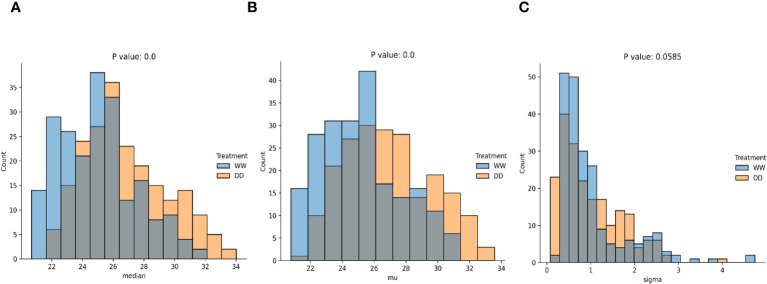
Statistical comparisons of data distributions (significant). Statistical comparison using the KS test of the median **(A)**, mean **(B)**, and variance **(C)** computed over the distributions of WW and DD leaves.

To enhance the mask M , the morphological operations of dilation, hole filling, and erosion, followed by a blob analysis, were performed, computing the connected components of the image. The final mask M is then selected as the region with the greatest number of contiguous pixels turned on. Some examples of leaf masks for different genotypes are shown in **Figure 5**. The pixel values in the region highlighted by the leaf masks are used to compute thermal features. As such, the temperature values are first statistically filtered removing the outliers, hence making the algorithm robust to small leaf mask misalignments. Then, a set of eight statistical thermal indicators are computed from raw thermal values, that is, mean, standard deviation, median, 25th and 75th percentiles, interquartile range, max, min, and temperature range.

The IP/ML software pipeline was developed and tested, and all the AI applications were run on a machine equipped with an Intel Core i9-11900K, 32 GB of RAM, and an Nvidia GeForce RTX 3080 GPU with 10 GB of RAM. The software was developed in Python 3.10, and the Scikit Image (Van der Walt et al., 2014) and Scikit Learn (Pedregosa et al., 2011) libraries were used.

## Results and discussion

### Statistical analyses

**Figure 6:**
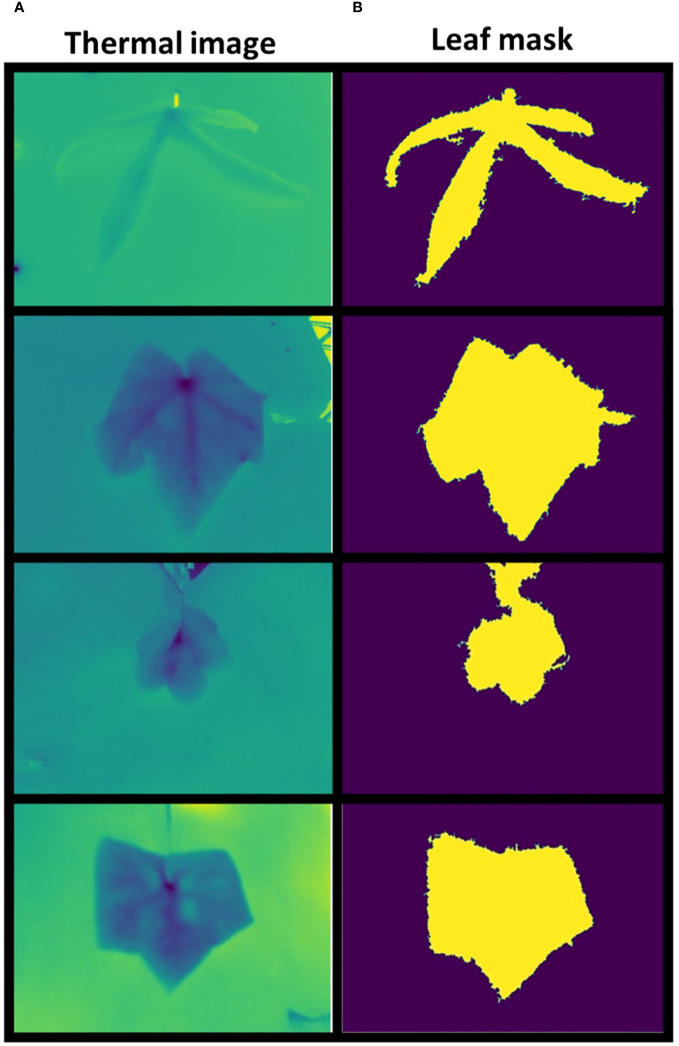
Leaf mask comparison. Examples of thermal images **(A)** and correspondent computed masks **(B)**.

The first step was to use the IP preprocessing techniques (**Figure S1**) to extract all the leaf masks from the raw thermal data along with the associated features. The algorithms used at this stage are non-parametric, meaning that they automatically tune the parameters after a preprocessing step of each thermal image, so that the leaf mask can be estimated (**Figure 6**) and the thermal features are extracted almost in real time. This step was mandatory for providing the baseline data to be used for the next steps of the pipeline.

The thermal features extracted from this initial data parsing step were then used as the basis for the ML processing software pipeline. For this, a first exploratory analysis was performed using a two-sample Kolmogorov–Smirnoff test on single features. This led to a comparison between well-watered leaves and droughted ones, aiming at identifying those features that were not sampled from the same statistical distribution. In other words, this test allowed the evaluation of features that were likely to be used to discriminate between WW and DD leaves.

**Figure 7:**
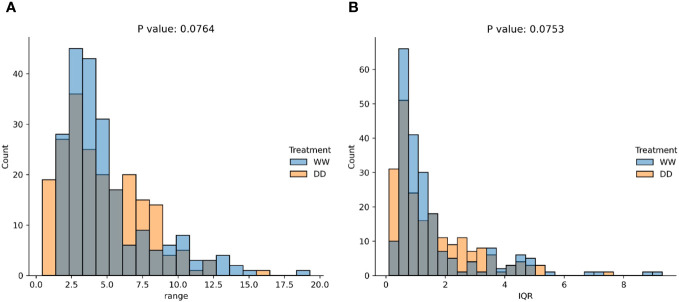
Statistical comparisons of data distributions (non-significant). Statistical comparison using the KS test of the data range **(A)** and interquartile range (IQR) **(B)** computed over the distributions of WW and DD leaves.

As no assumptions were made on the distribution for WW and DD leaves, two non-parametric distributions were anticipated. A comparison was performed using the median, mean, standard deviation, range, and interquartile range of each distribution (**Figures 5**, **7**). Median (**Figure 5A**), mean (**Figure 5B**), and standard deviation (**Figure 5C**) were statistically compared for the two distributions, and they all showed an extremely low *p*-value, below the standard threshold α=0.05 . As a consequence, the null hypothesis stating that data come from the same distribution could be rejected for these variables. When the data range and the interquartile range (IQR) were statistically compared (**Figures 5A, B**), the *p*-value was not lower than the standard threshold α , with *p* = 0.00764 and *p* = 0.0753, respectively. Hence, in this case, the null hypothesis could not be rejected, and we could not conclude that these quantities were drawn from different data distributions. From this statistical evaluation, we can assume that features related to the median, mean, and standard deviation of the values for the thermal features of the leaves can be effectively used to distinguish between WW and DD leaves. However, the range of the features and the interquartile range cannot be confidently considered during the evaluation since it cannot be concluded whether they are drawn from different distributions.

### Machine learning algorithms

A complete comparison between two different processing software pipelines was performed. Specifically, two different classifiers were trained to predict the plant treatment (DD or WW), that is, random forest (RF) and multilayer perceptron (MLP). The dataset used for the ML algorithms training, test, and validation was composed of 419 samples, 212 of them for WW leaves and 207 for DD leaves. Each sample is obtained by joining the automatically computed thermal features with the respective plant treatment (DD or WW), removing all the non-discriminating features from the dataset. The dataset subset split strategy was as follows: 75% of the samples (314) to compute the T subset (for the training and test) and 25% of the samples (105) to compute the V subset (for the validation). Each one of the ML algorithms was inserted in a pipeline, which first scaled each feature to match a normal distribution N(0,1) , namely, a distribution with zero-average and unitary standard deviation. A feature selection procedure was then performed using the mutual information criterion. Finally, the T subset was used to train and test the classifier using a random search for hyperparameter optimization and a K-fold cross-validation with k=10 . A summary of the results for optimization is shown in **Table 1** for both the RF and MLP pipelines.

**Table 1:** Hyperparameters selected for random forest and multilayer perceptron processing software pipelines.

Processing pipeline	Hyperparameter	Value	Description
RF pipeline (feature selection + random forest)	K	3	Number of the most relevant features selected according to the mutual information criterion
	Minimum samples per leaf	5	Minimum number of samples to determine whether a node of each tree in the decision forest can be marked as a terminal one (i.e., a leaf)
	Max depth	5	Maximum depth of each tree in the decision forest
MLP pipeline (feature selection + multilayer perceptron)	K	7	Number of the most relevant features selected according to the mutual information criterion
	Solver	ADAM	Optimization algorithm used during backpropagation
	Learning rate	Constant	Learning rate schedule used during backpropagation. In this case, constant means that no adaptive scheduling is used.
	Hidden layer sizes	50	Number of neurons used in the hidden layer of the multilayer perceptron

**Figure 8:**
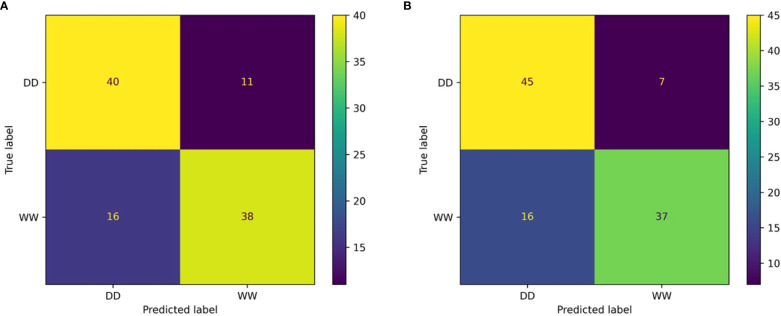
Confusion matrix outcomes. Confusion matrix for the random forest **(A)** and the multilayer perceptron **(B)** classifiers.

The resulting classification report (computed on the V subset) for the RF classifier showed weighted average values for precision and recall of 78% and 71%, respectively (**Table 2**). Overall, the weighted accuracy on a total support of 105 leaves across all genotypes was approximately 75%, meaning that the classifier was incorrect in predicting 25% of the original images during validation. Comparing the true labels of the leaves against the predicted labels using the RF classifier, 40 WW and 38 DD leaves across all genotypes were correctly predicted, while a total of 27 leaves were miscategorized (**Figure 8A**).

**Table 2:** Classification report for the random forest.

Class	Precision	Recall	F1 score	Weighted accuracy	Support
DD	0.78	0.71	0.74	0.75	51
WW	0.71	0.78	0.74	0.74	54

The MLP classifier showed slight overall improvements: the classifier achieved improved recall on DD leaves and precision on WW leaves, at the cost of lower values of precision and recall for DD and WW leaves, respectively (**Table 3**). However, there was an improvement in terms of the overall accuracy, increasing to 78%. It is important to highlight that for this second classifier, the data support was changed, although not significantly, due to the random generation process for the validation dataset used to ensure the generalization properties of the classifier. The higher overall accuracy of the MLP approach was reflected in the confusion matrix showing that the correct predictions across all 27 genotypes increased for both treatments (**Figure 8B**). Specifically, the MLP correctly categorized a total of 82 leaves between WW (37) and DD (45), compared with the 78 total of the RF classifier.

**Table 3:** Classification report for the multilayer perceptron.

Class	Precision	Recall	F1 score	Weighted accuracy	Support
DD	0.74	0.87	0.80	0.79	52
WW	0.84	0.70	0.76	0.78	53

Both the RF and MLP classifiers resulted in an accuracy greater than 70%, considering support data (105 leaves) pooled from 27 different genotypes for both WW and DD treatments, in mild and severe drought, corresponding to 4 and 14 days after the beginning of the progressive water withholding (**Figure 2**).

### Testing the physiological soundness of the AI analysis

Since the presented ML pipelines were built using the data from the entire panel of *Gossypium* under different degrees of water limitation, the accuracy results of the classifications can be considered in line with previous results (Solimani et al., 2023). The great genotypic diversity of the experimental cotton panel inevitably caused extreme variability in leaf size, plant and leaf architecture, and coloration (**Figure 1**). These diverse genotypes have already been reported to be indeed affected by their physiology resulting in a large spectrum of water status and drought response (Wendel et al., 2009; Wendel et al., 2010; Sreedasyam and Schmutz, 2019). This variability was clearly visible in the range of leaf temperatures captured already in WW conditions (**Figure 3**). For instance, the Delta Pine16 genotype showed a leaf temperature mean almost 5°C lower than *Tipo Chaco* in the same WW conditions, while two morphologically dissimilar genotypes, namely, *Dwarf Red Harrison* and *Siokara L23*—one with dark red/green, medium-size leaves and one with green, okra-like type of leaves—showed very similar leaf temperatures. It is known that leaf temperature is affected by changes in the microclimate at the canopy level and this can be somewhat variable in greenhouse conditions based on the spatial locations of the pots and on the time of the day (Beverly et al., 2020). However, drawing significant relationships between leaf temperature per se and genotypic variation was not the scope of the current work, and the diverse experimental panel was used as a robust testbed for the development of the novel IP/ML software pipeline for thermal data.

**Figure 9:**
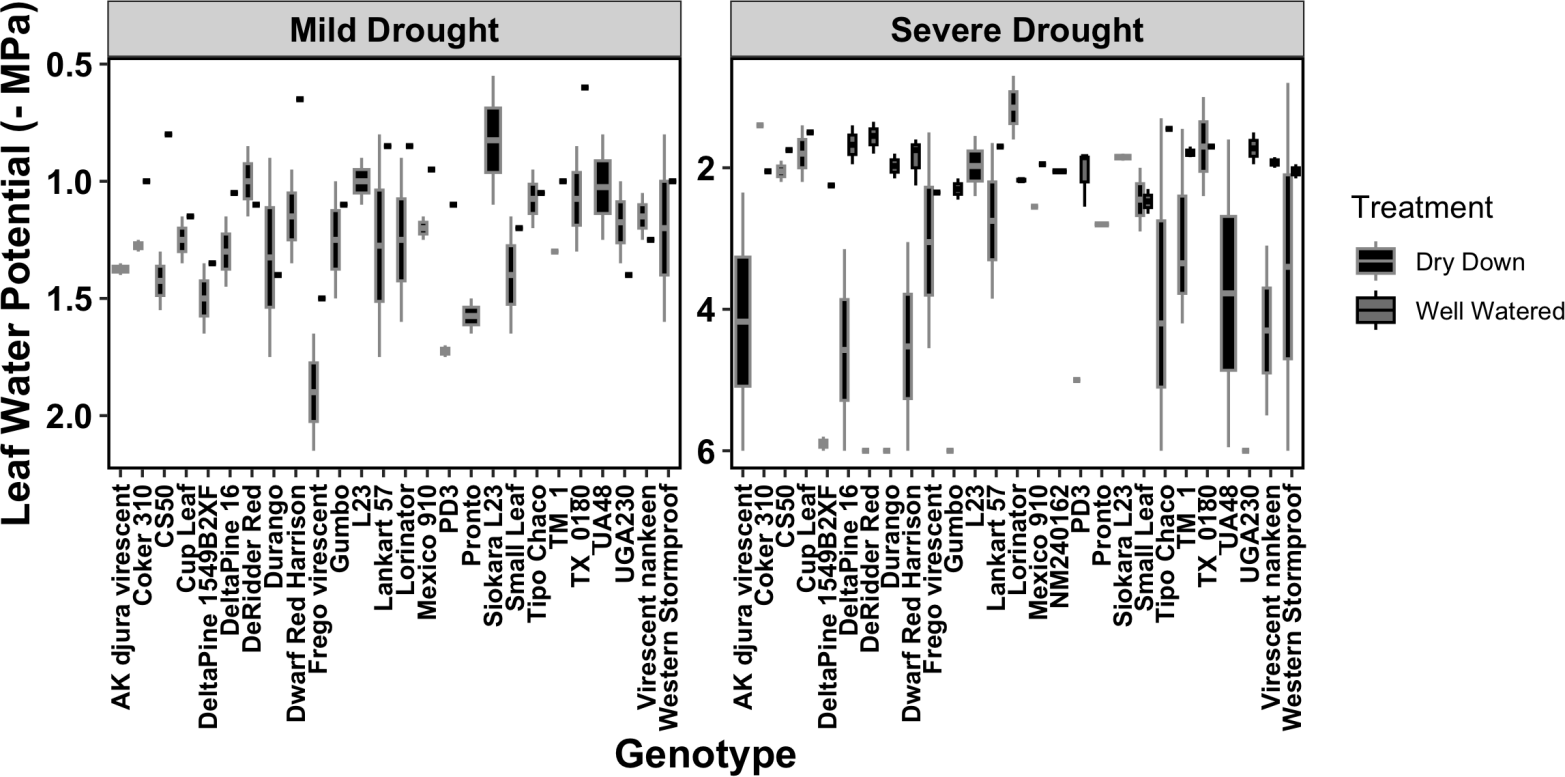
Leaf water potential across the 27 experimental genotypes. The distribution of leaf water potentials is observed across genotypes in both mild and severe drought. The well-watered plants (WW) are represented in gray and the plants under dry-down (DD) in black.

**Figure 10:**
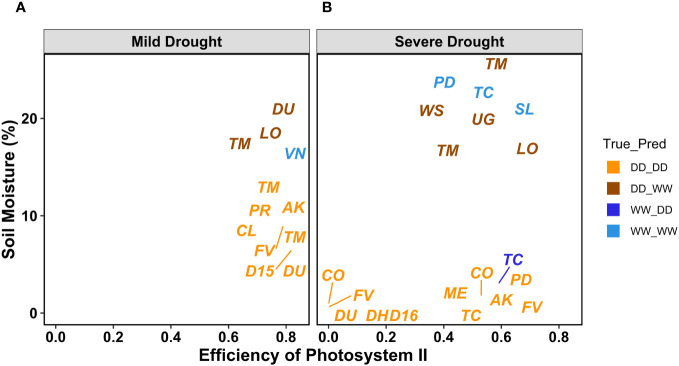
PSII efficiency and soil moisture as volumetric soil water content values for MLP outcomes. Labels refer to the true label compared with the MLP classification DD_DD (orange), DD_WW (brown), WW_DD (blue), and WW_WW (electric blue). A randomly chosen subset of leaves with the corresponding PSII efficiency and soil moisture from all genotypes and for both mild **(A)** and severe drought **(B)**.

To understand the misclassifications from the ML classifiers, we more closely analyzed the environmental and the volumetric soil water content associated with each image (**Supplementary Table S3**). First, we confirmed that the applied drought treatments caused a reduction of volumetric soil water content (%) for the DD plants compared with the WW, and this reduction was more evident under severe drought (**Figure S4**). As expected, the 27 genotypes responded differently to the progressive drought, with the most water-efficient genotypes like *Cup Leaf*, *L23*, and *Lorinator*, maintaining their leaf water potential closer to the WW value even under drought conditions (**Figure 9**). When comparing soil moisture and the efficiency of PSII from chlorophyll *a* fluorescence values for a random subset from all classified images, we found that the leaves wrongly classified by the ML pipeline also showed an outlier behavior in either one or both traits under both mild and severe drought conditions (**Figure 10**, **Figure S4**). For instance, under mild drought, the mislabeled genotypes (*TM1*, *Lorinator*, and *Durango*) were the ones that did not significantly decrease their soil moisture although they were sitting in the DD cohort of the panel, most likely due to microclimate variations in the greenhouse. The same validation with soil moisture was revealed for mislabeled plants under severe drought as well, and it similarly applied for plants sitting in the WW cohort, such as *Tipo Chaco* that for the true label of WW resulted in a predicted label of DD for the image pipeline (**Figure 8B**). While soil moisture seems to be sufficiently explanatory for the mislabeled leaves, the efficiency of PSII seems to be less correlated to the ML outcomes. While the randomly chosen DD leaves in severe drought showed high PSII efficiency, such as expected from their still relatively high soil moisture, the mislabeled *Tipo Chaco* sitting in the WW cohort was misclassified as DD by the ML pipelines even if it maintained a PSII efficiency of 0.55 (**Figure 10B**). Chlorophyll *a* fluorescence as the efficiency of PSII has previously been shown to follow drought response dynamics across different species (Guadagno et al., 2017), and this mismatch between soil moisture value and fluorescence might be due to a particular resistance of the photosynthetic machinery of this specific genotype to severe drought, which is not the focus of the presented work. This analysis of the software pipeline outcomes indicated soil moisture as a highly possible driver of the misclassification and the efficiency of PSII evidently being a less but still correlated physiological trait. From the physiological ground truthing, thermal imaging and the classifiers had lower than 25% and 22% mislabeled leaves (**Supplementary Table S3**) considering that the actual label was not meaningful of the actual treatment and/or physiological status of the plant.

## Conclusions

Our work confirmed the efficiency of thermal imaging data in detecting water limitations and the invaluable assistance of AI analysis in increasing the throughput of handheld IR cameras (Kamarudin and Ismail, 2022). Our results are suggestive of increased efficiency in the postprocessing of thermal data time even when extreme genotypic variation is present. In the utilized experimental panel, the spectrum of thermal features for different genotypes was in fact extremely variable even for WW samples. The presented classification becomes more meaningful considering that the support data for the ML application were coming from leaves exposed to two different levels of water limitation—aside from the WW control—triggering a large variety of physiological interplays across the 27 genotypes. Our leaf-level experimental approach coupled other physiological measurements to the thermal imaging, allowed us for further testing of the ML results. We found that mislabeled leaves also had a significantly different behavior in other means of plant water status such as soil water potential and content to partly account model errors. Overall, our study confirms that AI can be an incredible resource to optimize the throughput of handheld thermal cameras despite genotypic variation, extreme morphological and temperature features, and over a large combination of G × E, allowing for more generalized applications in water management across different geographical agricultural scenarios. In the future, we auspicate for the development of more targeted designs aimed to dissect the temporal progression of water limitation across different genotypes and its correlation with peculiar leaf venation types and architectures. Higher accuracy in thermal image classification will allow for the development of more complex ML pipelines, representing an essential aid in breeding and water management efforts,especially for globally relevant crop species like cotton.

## Data availability statement

The original contributions presented in the study are included in the article/**Supplementary Material**. Further inquiries can be directed to the corresponding author.

## Author contributions

VR: Conceptualization, Formal analysis, Methodology, Software, Visualization, Writing – original draft, Writing – review & editing, Investigation. AC: Formal analysis, Methodology, Software, Visualization, Writing – original draft, Investigation. BR: Data curation, Formal analysis, Investigation, Methodology, Visualization, Writing – original draft. CG: Conceptualization, Funding acquisition, Methodology, Project administration, Resources, Supervision, Validation, Visualization, Writing – original draft, Writing – review & editing.

## Supplementary material

Supplementary Figure S1 The processing image pipeline. Building blocks of the developed pipeline used in this work includes four computational steps: CSV parsing, pre-process, leaf mask computing, and thermal stats computing.

Supplementary Figure S2 Genotypes included in the experimental panel. All 27 genotypes included in the cotton experimental panel and their correspondent abbreviations used in during the experiment.

Supplementary Table S3 Single measurements of volumetric soil water content across all collected images.

Supplementary Figure S4 Volumetric soil water content across the 27 experimental genotypes. Distribution of leaf water potentials are observed across genotypes in both mild and severe drought. The well-watered plants (WW) are represented in grey and the plants under dry down (DD) in black.
