# Supplementary figures and images for: AI-assisted image analysis and physiological validation for progressive drought detection in a diverse panel of Gossypium hirsutum L
Source: Front Plant Sci. 2024 Feb 21;14:1305292. doi: 10.3389/fpls.2023.1305292 (PMC10915054; doi:10.3389/fpls.2023.1305292)

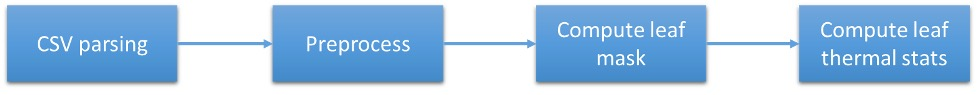

Supplement: Supplementary Figure S1 — The processing image pipeline. Building blocks of the developed pipeline used in this work includes four computational steps: CSV parsing, pre-process, leaf mask computing, and thermal stats computing. [file Image_1.tiff]

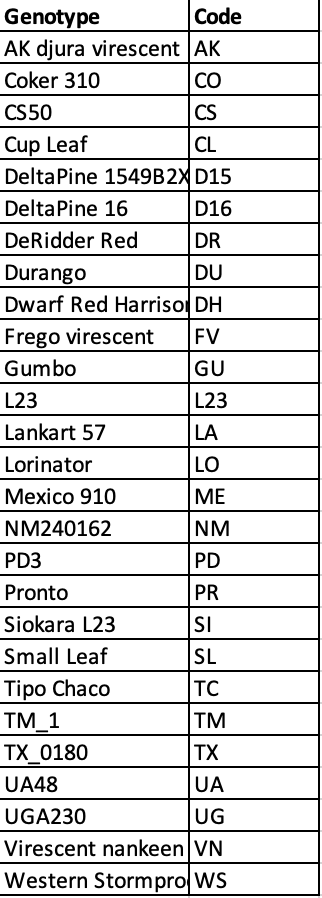

Supplement: Supplementary Figure S2 — Genotypes included in the experimental panel. All 27 genotypes included in the cotton experimental panel and their correspondent abbreviations used in during the experiment. [file Image_2.png]

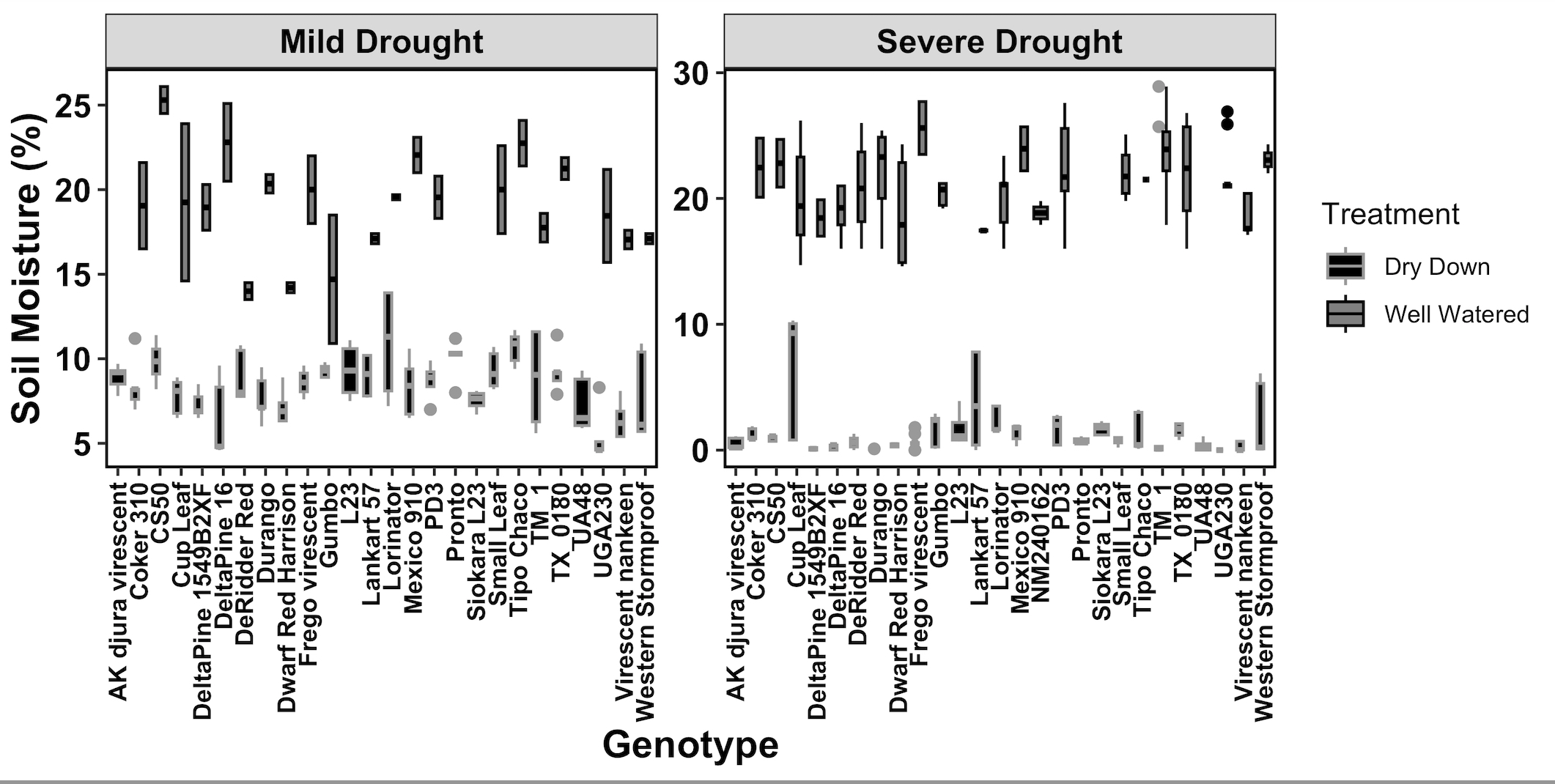

Supplement: Supplementary Figure S4 — Volumetric soil water content across the 27 experimental genotypes. Distribution of leaf water potentials are observed across genotypes in both mild and severe drought. The well-watered plants (WW) are represented in grey and the plants under dry down (DD) in black. [file Image_4.jpg]
