# Supplementary material for: AI-assisted image analysis and physiological validation for progressive drought detection in a diverse panel of Gossypium hirsutum L
Source: Front Plant Sci. 2024 Feb 21;14:1305292. doi: 10.3389/fpls.2023.1305292 (PMC10915054; doi:10.3389/fpls.2023.1305292)
Supplement: Supplementary Table S3 — Single measurements of volumetric soil water content across all collected images. [file Table_3.pdf]

Supplementary Table 3:

Time.Course	Time.of.Day	Treatment	Genotype	ID	Volumetric.Soil.Water.Content
Before Drought	Predawn	WW	AKDjuraVirescent	109	18.90
Before Drought	Predawn	WW	AKDjuraVirescent	136	23.00
Before Drought	Predawn	WW	Coker310	137	17.70
Before Drought	Predawn	WW	Coker310	29	19.80
Before Drought	Predawn	WW	CS50	30	20.30
Before Drought	Predawn	WW	CS50	3	28.50
Before Drought	Predawn	WW	CupLeaf	112	17.20
Before Drought	Predawn	WW	CupLeaf	58	22.90
Before Drought	Predawn	WW	DeltaPine154982XF	113	15.30
Before Drought	Predawn	WW	DeltaPine154982XF	59	19.00
Before Drought	Predawn	WW	DeltaPine16	6	19.10
Before Drought	Predawn	WW	DeltaPine16	60	19.60
Before Drought	Predawn	WW	DeRidderRed	115	21.50
Before Drought	Predawn	WW	DeRidderRed	142	25.80
Before Drought	Predawn	WW	Durango	62	23.80
Before Drought	Predawn	WW	Durango	143	25.10
Before Drought	Predawn	WW	DwarfRedHarrison	36	19.40
Before Drought	Predawn	WW	DwarfRedHarrison	90	20.80
Before Drought	Predawn	WW	FregoVirescent	91	15.90
Before Drought	Predawn	WW	FregoVirescent	10	20.10
Before Drought	Predawn	WW	Gumbo	119	19.30
Before Drought	Predawn	WW	Gumbo	65	24.70
Before Drought	Predawn	WW	L23	120	18.20
Before Drought	Predawn	WW	L23	93	18.50
Before Drought	Predawn	WW	Lankart57	67	20.40
Before Drought	Predawn	WW	Lankart57	148	25.80
Before Drought	Predawn	WW	Lorinator	14	16.20
Before Drought	Predawn	WW	Lorinator	122	20.40
Before Drought	Predawn	WW	Mexico910	42	22.10
Before Drought	Predawn	WW	Mexico910	150	23.20
Before Drought	Predawn	WW	PD3	17	18.80
Before Drought	Predawn	WW	PD3	98	20.40
Before Drought	Predawn	WW	Pronto	99	16.00
Before Drought	Predawn	WW	Pronto	45	24.00
Before Drought	Predawn	WW	SiokaraL23	154	16.50
Before Drought	Predawn	WW	SiokaraL23	19	22.50
Before Drought	Predawn	WW	SmallLeaf	74	23.10
Before Drought	Predawn	WW	SmallLeaf	47	28.70
Before Drought	Predawn	WW	Tipo_Chaco	102	19.40
Before Drought	Predawn	WW	Tipo_Chaco	75	22.70
Before Drought	Predawn	WW	TM_1	49	20.60
Before Drought	Predawn	WW	TM_1	157	24.00
Before Drought	Predawn	WW	TX_0180	104	13.20
Before Drought	Predawn	WW	TX_0180	50	18.60
Before Drought	Predawn	WW	UA48	78	18.70
Before Drought	Predawn	WW	UA48	159	24.20
Before Drought	Predawn	WW	UGA230	133	20.60
Before Drought	Predawn	WW	UGA230	160	21.40
Before Drought	Predawn	WW	VirescentNankeen	107	17.40
Before Drought	Predawn	WW	VirescentNankeen	26	22.70

Time.Course	Time.of.Day	Treatment	Genotype	ID	Volumetric.Soil.Water.Content
Before Drought	Predawn	WW	WesternStormproof	135	19.70
Before Drought	Predawn	WW	WesternStormproof	162	26.70
Before Drought	Midday	WW	AKDjuraVirescent	109	19.20
Before Drought	Midday	WW	AKDjuraVirescent	136	26.90
Before Drought	Midday	WW	Coker310	29	23.00
Before Drought	Midday	WW	Coker310	137	24.40
Before Drought	Midday	WW	CS50	30	22.50
Before Drought	Midday	WW	CS50	3	23.80
Before Drought	Midday	WW	CupLeaf	112	26.10
Before Drought	Midday	WW	CupLeaf	58	26.40
Before Drought	Midday	WW	DeltaPine154982XF	113	18.30
Before Drought	Midday	WW	DeltaPine154982XF	59	22.10
Before Drought	Midday	WW	DeltaPine16	6	20.90
Before Drought	Midday	WW	DeltaPine16	60	24.30
Before Drought	Midday	WW	DeRidderRed	115	21.40
Before Drought	Midday	WW	DeRidderRed	142	24.20
Before Drought	Midday	WW	Durango	143	27.30
Before Drought	Midday	WW	Durango	62	28.20
Before Drought	Midday	WW	DwarfRedHarrison	90	19.70
Before Drought	Midday	WW	DwarfRedHarrison	36	21.10
Before Drought	Midday	WW	FregoVirescent	10	17.30
Before Drought	Midday	WW	FregoVirescent	91	19.70
Before Drought	Midday	WW	Gumbo	119	19.90
Before Drought	Midday	WW	Gumbo	65	27.30
Before Drought	Midday	WW	L23	120	14.00
Before Drought	Midday	WW	L23	93	17.80
Before Drought	Midday	WW	Lankart57	148	25.70
Before Drought	Midday	WW	Lankart57	67	26.40
Before Drought	Midday	WW	Lorinator	122	21.00
Before Drought	Midday	WW	Lorinator	14	21.30
Before Drought	Midday	WW	Mexico910	42	24.20
Before Drought	Midday	WW	Mexico910	150	27.20
Before Drought	Midday	WW	PD3	98	18.00
Before Drought	Midday	WW	PD3	17	20.40
Before Drought	Midday	WW	Pronto	99	17.30
Before Drought	Midday	WW	Pronto	45	24.80
Before Drought	Midday	WW	SiokaraL23	154	22.50
Before Drought	Midday	WW	SiokaraL23	19	24.00
Before Drought	Midday	WW	SmallLeaf	74	18.90
Before Drought	Midday	WW	SmallLeaf	47	22.80
Before Drought	Midday	WW	Tipo__Chaco	75	22.80
Before Drought	Midday	WW	Tipo__Chaco	102	25.60
Before Drought	Midday	WW	TM__1	49	20.00
Before Drought	Midday	WW	TM__1	157	23.90
Before Drought	Midday	WW	TX__0180	104	16.00
Before Drought	Midday	WW	TX__0180	50	23.50
Before Drought	Midday	WW	UA48	78	20.00
Before Drought	Midday	WW	UA48	159	21.90
Before Drought	Midday	WW	UGA230	160	20.70
Before Drought	Midday	WW	UGA230	133	24.90
Before Drought	Midday	WW	VirescentNankeen	26	23.00
Before Drought	Midday	WW	VirescentNankeen	107	23.20

Time.Course	Time.of.Day	Treatment	Genotype	ID	Volumetric.Soil.Water.Content
Before Drought	Midday	WW	WesternStormproof	135	23.90
Before Drought	Midday	WW	WesternStormproof	162	26.20
Mild Drought	Predawn	DD	AKDjuraVirescent	109	13.90
Mild Drought	Predawn	DD	AKDjuraVirescent	136	20.60
Mild Drought	Predawn	DD	Coker310	29	7.40
Mild Drought	Predawn	DD	Coker310	56	8.20
Mild Drought	Predawn	DD	CS50	3	4.60
Mild Drought	Predawn	DD	CS50	30	7.60
Mild Drought	Predawn	DD	CupLeaf	85	9.90
Mild Drought	Predawn	DD	CupLeaf	112	15.70
Mild Drought	Predawn	DD	DeltaPine154982XF	59	8.30
Mild Drought	Predawn	DD	DeltaPine154982XF	113	16.80
Mild Drought	Predawn	DD	DeltaPine16	6	5.10
Mild Drought	Predawn	DD	DeltaPine16	60	8.50
Mild Drought	Predawn	DD	DeRidderRed	7	5.70
Mild Drought	Predawn	DD	DeRidderRed	115	16.90
Mild Drought	Predawn	DD	Durango	35	7.80
Mild Drought	Predawn	DD	Durango	62	8.70
Mild Drought	Predawn	DD	DwarfRedHarrison	36	7.90
Mild Drought	Predawn	DD	DwarfRedHarrison	90	10.30
Mild Drought	Predawn	DD	FregoVirescent	10	6.10
Mild Drought	Predawn	DD	FregoVirescent	64	8.80
Mild Drought	Predawn	DD	Gumbo	65	8.90
Mild Drought	Predawn	DD	Gumbo	119	17.40
Mild Drought	Predawn	DD	L23	93	10.50
Mild Drought	Predawn	DD	L23	120	17.60
Mild Drought	Predawn	DD	Lankart57	67	9.00
Mild Drought	Predawn	DD	Lankart57	148	21.20
Mild Drought	Predawn	DD	Lorinator	14	6.40
Mild Drought	Predawn	DD	Lorinator	122	18.60
Mild Drought	Predawn	DD	Mexico910	42	8.00
Mild Drought	Predawn	DD	Mexico910	150	21.90
Mild Drought	Predawn	DD	PD3	17	6.50
Mild Drought	Predawn	DD	PD3	98	10.70
Mild Drought	Predawn	DD	Pronto	45	8.10
Mild Drought	Predawn	DD	Pronto	99	10.90
Mild Drought	Predawn	DD	SiokaraL23	19	6.50
Mild Drought	Predawn	DD	SiokaraL23	154	22.00
Mild Drought	Predawn	DD	SmallLeaf	20	6.50
Mild Drought	Predawn	DD	SmallLeaf	47	8.10
Mild Drought	Predawn	DD	Tipo_Chaco	21	6.90
Mild Drought	Predawn	DD	Tipo_Chaco	102	11.20
Mild Drought	Predawn	DD	TM_1	22	7.00
Mild Drought	Predawn	DD	TM_1	157	23.90
Mild Drought	Predawn	DD	TX_0180	50	8.20
Mild Drought	Predawn	DD	TX_0180	104	11.40
Mild Drought	Predawn	DD	UA48	78	9.40
Mild Drought	Predawn	DD	UA48	159	24.10
Mild Drought	Predawn	DD	UGA230	79	9.40
Mild Drought	Predawn	DD	UGA230	133	19.80
Mild Drought	Predawn	DD	VirescentNankeen	26	7.20
Mild Drought	Predawn	DD	VirescentNankeen	80	9.60

Time.Course	Time.of.Day	Treatment	Genotype	ID	Volumetric.Soil.Water.Content
Mild Drought	Predawn	DD	WesternStormproof	27	7.40
Mild Drought	Predawn	DD	WesternStormproof	135	20.30
Mild Drought	Predawn	WW	Coker310	110	14.50
Mild Drought	Predawn	WW	CS50	84	9.70
Mild Drought	Predawn	WW	CupLeaf	31	7.60
Mild Drought	Predawn	WW	DeltaPine154982XF	5	4.60
Mild Drought	Predawn	WW	DeltaPine16	87	10.20
Mild Drought	Predawn	WW	DeRidderRed	34	7.70
Mild Drought	Predawn	WW	Durango	8	6.00
Mild Drought	Predawn	WW	DwarfRedHarrison	117	17.40
Mild Drought	Predawn	WW	FregoVirescent	145	20.90
Mild Drought	Predawn	WW	Gumbo	38	7.90
Mild Drought	Predawn	WW	Lankart57	121	18.30
Mild Drought	Predawn	WW	Lorinator	95	10.60
Mild Drought	Predawn	WW	Mexico910	69	9.00
Mild Drought	Predawn	WW	PD3	71	9.10
Mild Drought	Predawn	WW	SmallLeaf	101	10.90
Mild Drought	Predawn	WW	Tipo_Chaco	75	9.20
Mild Drought	Predawn	WW	TM_1	103	11.30
Mild Drought	Predawn	WW	TX_0180	77	9.30
Mild Drought	Predawn	WW	UGA230	160	25.10
Mild Drought	Predawn	WW	VirescentNankeen	107	11.60
Mild Drought	Predawn	WW	WesternStormproof	108	13.90
Mild Drought	Midday	DD	AKDjuraVirescent	109	14.50
Mild Drought	Midday	DD	AKDjuraVirescent	136	20.80
Mild Drought	Midday	DD	Coker310	29	7.50
Mild Drought	Midday	DD	Coker310	56	8.20
Mild Drought	Midday	DD	CS50	3	4.60
Mild Drought	Midday	DD	CS50	30	7.50
Mild Drought	Midday	DD	CupLeaf	85	9.90
Mild Drought	Midday	DD	CupLeaf	112	16.50
Mild Drought	Midday	DD	DeltaPine154982XF	59	8.40
Mild Drought	Midday	DD	DeltaPine154982XF	113	16.50
Mild Drought	Midday	DD	DeltaPine16	6	5.40
Mild Drought	Midday	DD	DeltaPine16	60	8.60
Mild Drought	Midday	DD	DeRidderRed	7	5.60
Mild Drought	Midday	DD	DeRidderRed	115	16.80
Mild Drought	Midday	DD	Durango	35	7.80
Mild Drought	Midday	DD	Durango	62	8.80
Mild Drought	Midday	DD	DwarfRedHarrison	36	7.90
Mild Drought	Midday	DD	DwarfRedHarrison	90	10.30
Mild Drought	Midday	DD	FregoVirescent	10	6.20
Mild Drought	Midday	DD	FregoVirescent	64	8.90
Mild Drought	Midday	DD	Gumbo	65	8.90
Mild Drought	Midday	DD	Gumbo	119	17.60
Mild Drought	Midday	DD	L23	93	10.40
Mild Drought	Midday	DD	L23	120	18.00
Mild Drought	Midday	DD	Lankart57	67	8.90
Mild Drought	Midday	DD	Lankart57	148	21.40
Mild Drought	Midday	DD	Lorinator	14	6.30
Mild Drought	Midday	DD	Lorinator	122	19.40
Mild Drought	Midday	DD	Mexico910	42	8.00

Time.Course	Time.of.Day	Treatment	Genotype	ID	Volumetric.Soil.Water.Content
Mild Drought	Midday	DD	Mexico910	150	21.60
Mild Drought	Midday	DD	PD3	17	6.50
Mild Drought	Midday	DD	PD3	98	10.80
Mild Drought	Midday	DD	Pronto	45	8.00
Mild Drought	Midday	DD	Pronto	99	10.90
Mild Drought	Midday	DD	SiokaraL23	19	6.50
Mild Drought	Midday	DD	SiokaraL23	154	22.60
Mild Drought	Midday	DD	SmallLeaf	20	6.70
Mild Drought	Midday	DD	SmallLeaf	47	8.10
Mild Drought	Midday	DD	Tipo_Chaco	21	6.90
Mild Drought	Midday	DD	Tipo_Chaco	102	11.20
Mild Drought	Midday	DD	TM_1	22	7.00
Mild Drought	Midday	DD	TM_1	157	23.10
Mild Drought	Midday	DD	TX_0180	50	8.20
Mild Drought	Midday	DD	TX_0180	104	11.60
Mild Drought	Midday	DD	UA48	78	9.40
Mild Drought	Midday	DD	UA48	159	24.50
Mild Drought	Midday	DD	UGA230	79	9.50
Mild Drought	Midday	DD	UGA230	133	19.70
Mild Drought	Midday	DD	VirescentNankeen	26	7.10
Mild Drought	Midday	DD	VirescentNankeen	80	9.60
Mild Drought	Midday	DD	WesternStormproof	27	7.40
Mild Drought	Midday	DD	WesternStormproof	135	20.50
Mild Drought	Midday	WW	Coker310	110	14.60
Mild Drought	Midday	WW	CS50	84	9.80
Mild Drought	Midday	WW	CupLeaf	31	7.60
Mild Drought	Midday	WW	DeltaPine154982XF	5	4.80
Mild Drought	Midday	WW	DeltaPine16	87	10.30
Mild Drought	Midday	WW	DeRidderRed	34	7.70
Mild Drought	Midday	WW	Durango	8	5.90
Mild Drought	Midday	WW	DwarfRedHarrison	117	17.40
Mild Drought	Midday	WW	FregoVirescent	145	21.00
Mild Drought	Midday	WW	Gumbo	38	7.90
Mild Drought	Midday	WW	Lankart57	121	18.50
Mild Drought	Midday	WW	Lorinator	95	10.60
Mild Drought	Midday	WW	Mexico910	69	9.10
Mild Drought	Midday	WW	PD3	71	9.20
Mild Drought	Midday	WW	SmallLeaf	101	11.10
Mild Drought	Midday	WW	Tipo_Chaco	75	9.30
Mild Drought	Midday	WW	TM_1	103	11.40
Mild Drought	Midday	WW	TX_0180	77	9.30
Mild Drought	Midday	WW	UGA230	160	26.10
Mild Drought	Midday	WW	VirescentNankeen	107	11.70
Mild Drought	Midday	WW	WesternStormproof	108	13.50
Severe Drought	Predawn	DD	AKDjuraVirescent	109	0.20
Severe Drought	Predawn	DD	AKDjuraVirescent	136	0.80
Severe Drought	Predawn	DD	Coker310	29	0.90
Severe Drought	Predawn	DD	Coker310	56	1.70
Severe Drought	Predawn	DD	CS50	3	1.10
Severe Drought	Predawn	DD	CS50	30	0.60
Severe Drought	Predawn	DD	CupLeaf	4	14.70
Severe Drought	Predawn	DD	CupLeaf	85	0.70

Time.Course	Time.of.Day	Treatment	Genotype	ID	Volumetric.Soil.Water.Content
Severe Drought	Predawn	DD	CupLeaf	112	10.30
Severe Drought	Predawn	DD	DeltaPine154982XF	59	0.01
Severe Drought	Predawn	DD	DeltaPine154982XF	113	0.20
Severe Drought	Predawn	DD	DeltaPine16	6	0.40
Severe Drought	Predawn	DD	DeltaPine16	60	0.40
Severe Drought	Predawn	DD	DeRidderRed	7	1.30
Severe Drought	Predawn	DD	DeRidderRed	115	0.01
Severe Drought	Predawn	DD	Durango	35	0.01
Severe Drought	Predawn	DD	Durango	62	0.01
Severe Drought	Predawn	DD	DwarfRedHarrison	36	0.20
Severe Drought	Predawn	DD	DwarfRedHarrison	90	0.50
Severe Drought	Predawn	DD	FregoVirescent	10	0.50
Severe Drought	Predawn	DD	FregoVirescent	64	0.60
Severe Drought	Predawn	DD	FregoVirescent	91	1.80
Severe Drought	Predawn	DD	Gumbo	65	0.10
Severe Drought	Predawn	DD	Gumbo	119	2.40
Severe Drought	Predawn	DD	L23	93	2.20
Severe Drought	Predawn	DD	L23	120	1.10
Severe Drought	Predawn	DD	Lankart57	67	7.80
Severe Drought	Predawn	DD	Lankart57	148	0.01
Severe Drought	Predawn	DD	Lorinator	14	3.50
Severe Drought	Predawn	DD	Lorinator	122	1.60
Severe Drought	Predawn	DD	Mexico910	42	1.80
Severe Drought	Predawn	DD	Mexico910	150	0.90
Severe Drought	Predawn	DD	PD3	17	0.40
Severe Drought	Predawn	DD	PD3	98	2.00
Severe Drought	Predawn	DD	Pronto	45	0.40
Severe Drought	Predawn	DD	Pronto	99	0.90
Severe Drought	Predawn	DD	SiokaraL23	19	1.80
Severe Drought	Predawn	DD	SiokaraL23	154	1.10
Severe Drought	Predawn	DD	SmallLeaf	20	1.00
Severe Drought	Predawn	DD	SmallLeaf	47	0.20
Severe Drought	Predawn	DD	Tipo_Chaco	21	3.00
Severe Drought	Predawn	DD	Tipo_Chaco	48	0.20
Severe Drought	Predawn	DD	Tipo_Chaco	102	0.40
Severe Drought	Predawn	DD	TM_1	22	0.01
Severe Drought	Predawn	DD	TM_1	130	25.70
Severe Drought	Predawn	DD	TM_1	157	0.30
Severe Drought	Predawn	DD	TX_0180	50	0.80
Severe Drought	Predawn	DD	TX_0180	104	2.10
Severe Drought	Predawn	DD	UA48	78	1.10
Severe Drought	Predawn	DD	UA48	159	0.01
Severe Drought	Predawn	DD	UGA230	79	0.01
Severe Drought	Predawn	DD	UGA230	133	0.01
Severe Drought	Predawn	DD	VirescentNankeen	26	0.01
Severe Drought	Predawn	DD	VirescentNankeen	80	0.80
Severe Drought	Predawn	DD	WesternStormproof	27	5.30
Severe Drought	Predawn	DD	WesternStormproof	135	0.10
Severe Drought	Predawn	WW	Coker310	110	20.10
Severe Drought	Predawn	WW	CS50	84	20.90
Severe Drought	Predawn	WW	CupLeaf	58	24.20
Severe Drought	Predawn	WW	DeltaPine154982XF	5	17.00

Time.Course	Time.of.Day	Treatment	Genotype	ID	Volumetric.Soil.Water.Content
Severe Drought	Predawn	WW	DeltaPine16	87	17.90
Severe Drought	Predawn	WW	DeltaPine16	114	16.00
Severe Drought	Predawn	WW	DeRidderRed	34	16.50
Severe Drought	Predawn	WW	DeRidderRed	88	20.80
Severe Drought	Predawn	WW	DeRidderRed	142	16.00
Severe Drought	Predawn	WW	Durango	8	23.30
Severe Drought	Predawn	WW	Durango	89	16.00
Severe Drought	Predawn	WW	DwarfRedHarrison	63	20.80
Severe Drought	Predawn	WW	DwarfRedHarrison	117	15.00
Severe Drought	Predawn	WW	DwarfRedHarrison	144	22.40
Severe Drought	Predawn	WW	FregoVirescent	145	23.50
Severe Drought	Predawn	WW	Gumbo	11	20.20
Severe Drought	Predawn	WW	Gumbo	38	19.20
Severe Drought	Predawn	WW	Lankart57	94	11.80
Severe Drought	Predawn	WW	Lankart57	121	17.40
Severe Drought	Predawn	WW	Lorinator	41	21.80
Severe Drought	Predawn	WW	Lorinator	95	16.00
Severe Drought	Predawn	WW	Lorinator	149	18.10
Severe Drought	Predawn	WW	Mexico910	69	22.20
Severe Drought	Predawn	WW	NM240162	70	17.90
Severe Drought	Predawn	WW	PD3	44	16.00
Severe Drought	Predawn	WW	PD3	71	20.60
Severe Drought	Predawn	WW	PD3	125	24.90
Severe Drought	Predawn	WW	SmallLeaf	74	20.60
Severe Drought	Predawn	WW	SmallLeaf	101	19.80
Severe Drought	Predawn	WW	Tipo_Chaco	75	21.50
Severe Drought	Predawn	WW	TM_1	49	25.40
Severe Drought	Predawn	WW	TM_1	76	25.30
Severe Drought	Predawn	WW	TM_1	103	17.90
Severe Drought	Predawn	WW	TX_0180	23	16.00
Severe Drought	Predawn	WW	TX_0180	77	22.40
Severe Drought	Predawn	WW	UGA230	52	25.90
Severe Drought	Predawn	WW	UGA230	160	21.10
Severe Drought	Predawn	WW	VirescentNankeen	53	17.10
Severe Drought	Predawn	WW	VirescentNankeen	107	17.60
Severe Drought	Predawn	WW	WesternStormproof	108	22.00
Severe Drought	Predawn	WW	WesternStormproof	162	23.40
Severe Drought	Midday	DD	AKDjuraVirescent	109	0.20
Severe Drought	Midday	DD	AKDjuraVirescent	136	1.10
Severe Drought	Midday	DD	Coker310	29	0.90
Severe Drought	Midday	DD	Coker310	56	1.90
Severe Drought	Midday	DD	CS50	3	1.30
Severe Drought	Midday	DD	CS50	30	0.70
Severe Drought	Midday	DD	CupLeaf	4	17.10
Severe Drought	Midday	DD	CupLeaf	85	0.80
Severe Drought	Midday	DD	CupLeaf	112	9.30
Severe Drought	Midday	DD	DeltaPine154982XF	59	0.20
Severe Drought	Midday	DD	DeltaPine154982XF	113	0.01
Severe Drought	Midday	DD	DeltaPine16	6	0.01
Severe Drought	Midday	DD	DeltaPine16	60	0.01
Severe Drought	Midday	DD	DeRidderRed	7	0.60
Severe Drought	Midday	DD	DeRidderRed	115	0.50

Time.Course	Time.of.Day	Treatment	Genotype	ID	Volumetric.Soil.Water.Content
Severe Drought	Midday	DD	Durango	35	0.10
Severe Drought	Midday	DD	Durango	62	0.01
Severe Drought	Midday	DD	DwarfRedHarrison	36	0.60
Severe Drought	Midday	DD	DwarfRedHarrison	90	0.40
Severe Drought	Midday	DD	FregoVirescent	10	0.01
Severe Drought	Midday	DD	FregoVirescent	64	0.60
Severe Drought	Midday	DD	FregoVirescent	91	1.30
Severe Drought	Midday	DD	Gumbo	65	0.30
Severe Drought	Midday	DD	Gumbo	119	2.90
Severe Drought	Midday	DD	L23	93	3.90
Severe Drought	Midday	DD	L23	120	0.70
Severe Drought	Midday	DD	Lankart57	67	3.50
Severe Drought	Midday	DD	Lankart57	148	0.40
Severe Drought	Midday	DD	Lorinator	14	1.60
Severe Drought	Midday	DD	Lorinator	122	1.40
Severe Drought	Midday	DD	Mexico910	42	1.90
Severe Drought	Midday	DD	Mexico910	150	0.30
Severe Drought	Midday	DD	PD3	17	0.40
Severe Drought	Midday	DD	PD3	98	2.80
Severe Drought	Midday	DD	Pronto	45	0.60
Severe Drought	Midday	DD	Pronto	99	1.10
Severe Drought	Midday	DD	SiokaraL23	19	2.30
Severe Drought	Midday	DD	SiokaraL23	154	1.20
Severe Drought	Midday	DD	SmallLeaf	20	1.00
Severe Drought	Midday	DD	SmallLeaf	47	0.70
Severe Drought	Midday	DD	Tipo_Chaco	21	3.20
Severe Drought	Midday	DD	Tipo_Chaco	48	0.10
Severe Drought	Midday	DD	Tipo_Chaco	102	0.30
Severe Drought	Midday	DD	TM_1	22	0.01
Severe Drought	Midday	DD	TM_1	130	28.90
Severe Drought	Midday	DD	TM_1	157	0.10
Severe Drought	Midday	DD	TX_0180	50	1.30
Severe Drought	Midday	DD	TX_0180	104	2.10
Severe Drought	Midday	DD	UA48	78	0.50
Severe Drought	Midday	DD	UA48	159	0.01
Severe Drought	Midday	DD	UGA230	79	0.01
Severe Drought	Midday	DD	UGA230	133	0.01
Severe Drought	Midday	DD	VirescentNankeen	26	0.01
Severe Drought	Midday	DD	VirescentNankeen	80	0.70
Severe Drought	Midday	DD	WesternStormproof	27	6.10
Severe Drought	Midday	DD	WesternStormproof	135	0.20
Severe Drought	Midday	WW	Coker310	110	24.80
Severe Drought	Midday	WW	CS50	84	24.70
Severe Drought	Midday	WW	CupLeaf	31	23.30
Severe Drought	Midday	WW	CupLeaf	58	26.20
Severe Drought	Midday	WW	DeltaPine154982XF	5	19.90
Severe Drought	Midday	WW	DeltaPine16	87	21.00
Severe Drought	Midday	WW	DeltaPine16	114	20.60
Severe Drought	Midday	WW	DeRidderRed	34	19.80
Severe Drought	Midday	WW	DeRidderRed	88	26.00
Severe Drought	Midday	WW	DeRidderRed	142	23.70
Severe Drought	Midday	WW	Durango	8	25.40

Time.Course	Time.of.Day	Treatment	Genotype	ID	Volumetric.Soil.Water.Content
Severe Drought	Midday	WW	Durango	89	18.90
Severe Drought	Midday	WW	DwarfRedHarrison	63	24.30
Severe Drought	Midday	WW	DwarfRedHarrison	117	14.60
Severe Drought	Midday	WW	DwarfRedHarrison	144	24.20
Severe Drought	Midday	WW	FregoVirescent	145	27.70
Severe Drought	Midday	WW	Gumbo	11	21.30
Severe Drought	Midday	WW	Gumbo	38	21.20
Severe Drought	Midday	WW	Lankart57	94	18.80
Severe Drought	Midday	WW	Lankart57	121	17.50
Severe Drought	Midday	WW	Lorinator	41	23.40
Severe Drought	Midday	WW	Lorinator	95	21.20
Severe Drought	Midday	WW	Lorinator	149	21.10
Severe Drought	Midday	WW	Mexico910	69	25.70
Severe Drought	Midday	WW	NM240162	70	19.80
Severe Drought	Midday	WW	PD3	44	21.70
Severe Drought	Midday	WW	PD3	71	27.60
Severe Drought	Midday	WW	PD3	125	22.10
Severe Drought	Midday	WW	SmallLeaf	74	25.10
Severe Drought	Midday	WW	SmallLeaf	101	22.90
Severe Drought	Midday	WW	Tipo_Chaco	75	21.50
Severe Drought	Midday	WW	TM_1	49	23.90
Severe Drought	Midday	WW	TM_1	76	28.90
Severe Drought	Midday	WW	TM_1	103	22.20
Severe Drought	Midday	WW	TX_0180	23	17.90
Severe Drought	Midday	WW	TX_0180	77	26.80
Severe Drought	Midday	WW	UGA230	52	26.90
Severe Drought	Midday	WW	UGA230	160	20.90
Severe Drought	Midday	WW	VirescentNankeen	53	17.40
Severe Drought	Midday	WW	VirescentNankeen	107	20.40
Severe Drought	Midday	WW	WesternStormproof	108	24.30
Severe Drought	Midday	WW	WesternStormproof	162	22.70
